# Metabolome and Metagenome Signatures Underlying the Differential Resistance of *Percocypris pingi*, Crucian Carp, and Yellow Catfish to *Ichthyophthirius multifiliis* Infection

**DOI:** 10.3390/biology14111546

**Published:** 2025-11-04

**Authors:** Yi Liu, Jiang Xie, Yang He, Qingchao Shi, Quan Gong, Weihong Zhao, Chuanjie Qin, Chuang Zhou

**Affiliations:** 1 Fishes Conservation and Utilization in the Upper Reaches of the Yangtze River Key Laboratory of Sichuan Province, College of Fisheries, Neijiang Normal University, Neijiang 641100, China; liuyi_shz@163.com (Y.L.); xiejianggz@163.com (J.X.); he_yang_yang@126.com (Y.H.); gaoyisqc@126.com (Q.S.); 2 Fisheries Research Institute, Sichuan Academy of Agricultural Sciences (Sichuan Fisheries Research Institute), Chengdu 611731, China; admiral671@163.com; 3 School of Marine and Bioengineering, Yancheng Institute of Technology, Yancheng 224051, China; zhaoweihong97@163.com; 4 Key Laboratory of Bio-Resources and Eco-Environment of Ministry of Education, College of Life Sciences, Sichuan University, Chengdu 610065, China; 5 Observation and Research Station of Sichuan Province of Fish Resources and Environment in Upper Reaches of the Yangtze River, College of Life Sciences, Sichuan University, Chengdu 610065, China

**Keywords:** white spot, disease resistance, skin microbiome, antioxidant capacity, immunity, *Candidatus Megaira*

## Abstract

*Ichthyophthirius multifiliis* poses a significant threat to global aquaculture, yet some fish species exhibit remarkable resistance. This study employed a combined LC-MS-based metabolomics and 16S rRNA gene sequencing approach to investigate the intrinsic mechanisms underlying the differential susceptibility of *Percocypris pingi*, crucian carp, and yellow catfish. Our results revealed distinct skin molecular and microbial profiles in *P. pingi* associated with its enhanced resilience. Metabolomic analysis identified a significant upregulation of key antioxidants (L-Glutathione reduced, L-Glutathione oxidized, L-Cysteine-glutathione gisulfide, Uric acid, Histamine, N-Acetylhistamine, and scorbic acid) in *P. pingi*, most notably L-Glutathione reduced, which was 31- and 59-fold higher than in yellow catfish and crucian carp, respectively. Functional enrichment further highlighted the critical role of enhanced antioxidant capacity (centered on glutathione metabolism) and immune/inflammatory responses in the resistance to *I. multifiliis* of *P. pingi*. Concurrently, skin microbiome analysis showed that *P. pingi* hosted a microbial community distinct from the other two species, with significantly higher α-diversity. Notably, *P. pingi* skin was significantly depleted of the parasitic bacteria *Candidatus_Megaira* and *Candidatus_Midichloria*, which were highly abundant in the susceptible species. Furthermore, predicted metagenomic functions indicated that *P. pingi*’s microbiota was enriched in fundamental metabolic pathways, whereas the microbiota of crucian carp and yellow catfish was skewed towards disease- and immune-related pathways. In conclusion, our findings demonstrate that the superior resistance of *P. pingi* to *I. multifiliis* is likely conferred by a synergistic effect of a robust skin antioxidant capacity (primarily driven by glutathione) and a protective skin microbiome that excludes specific parasites. This study provides novel insights into the multi-faceted mechanisms of disease resistance in fish.

## Simple Summary

Some fish species are naturally more resistant to the parasitic disease “white spot” caused by *Ichthyophthirius multifiliis*, which is a major problem in fish farming. To understand why, we studied three fish species: the resistant *Percocypris pingi* and the more susceptible crucian carp and yellow catfish. We discovered that the skin of the resistant *P. pingi* has a unique combination of features. It contains very high levels of protective antioxidant molecules, especially glutathione, which was dozens of times more abundant than in the other species. At the same time, the community of microbes on its skin is different and more diverse, notably lacking certain parasitic bacteria that are common on the susceptible fish. We conclude that the remarkable disease resistance of *P. pingi* is due to this powerful one-two punch: a strong antioxidant system working together with a protective skin microbiome. These findings help us understand the natural defense mechanisms that fish can use to fight disease.

## 1. Introduction

*Ichthyophthirius multifiliis*, commonly known as ich, is a ciliated protozoan ectoparasite of freshwater fish, belonging to the phylum Ciliophora, class Oligohymenophorea, family Ichthyophthiriidae, and genus *Ichthyophthirius*. After the larval stage of *I. multifiliis* invades the fish’s skin, it feeds on mucus and epithelial cells for nutrition. Propelled by ciliary movement, the parasite migrates beneath the skin or resides in localized areas, inducing tissue hyperplasia and excessive mucus production [1]. This leads to the appearance of visible white spots on the fish’s body, a condition known as ichthyophthiriasis or white spot disease. Due to its simple life cycle, low host specificity, and wide distribution, *I. multifiliis* causes high mortality in infected fish. It threatens nearly all freshwater fish species worldwide, inflicting significant economic losses on the global aquaculture industry and posing risks to wild populations. Consequently, ichthyophthiriasis is often referred to as the “cancer” of fish [2,3]. *I. multifiliis* infestations have imposed substantial economic burdens on the aquaculture industry, and annual losses of around $140 million for European trout farmers [4].

Among various strategies for controlling parasitic diseases, breeding disease-resistant varieties is the most effective, safe, and environmentally friendly approach. The discovery of species with resistant traits and the elucidation of underlying resistance mechanisms are prerequisites for genetic breeding programs. Susceptibility and resistance to *I. multifiliis* vary among different fish species and even among different genetic strains within the same species. Clayton and Price found that two goodeid species derived from wild populations, *Ameca splendens* and *Ilyodon xantusi*, were more susceptible to *I. multifiliis* than two poeciliid species with longer domestication histories, *Xiphophorus maculatus* and *Xiphophorus variatus* [5]. Furthermore, significant intraspecific differences were observed among *X. maculatus*, *X. variatus*, and their hybrids, with hybrid individuals exhibiting significantly lower infection levels than their parents after removing environmental influences, suggesting that genetic background plays an important role in determining resistance [6]. Other studies have shown that *Spinibarbus hollandi* possesses stronger immunity against *I. multifiliis* than grass carp (*Ctenopharyngodon idella*) [7]. During over a decade of resource conservation and artificial domestication practices, our research team observed that *Percocypris pingi* is highly resistant to *I. multifiliis* infection. No infections were detected from the larval, juvenile, sub-adult, or adult stages, even during severe outbreaks or when cohabiting with infected fish [8]. The susceptibility and resistance of *P. pingi* to four other freshwater fish species—southern catfish (*Silurus meridionalis*), grass carp, *Schizothorax prenanti*, and topmouth culter (*Culter alburnus*)—demonstrated that *P. pingi* restricts the parasitism and growth of *I. multifiliis* trophonts [9]. Building on the high resistance of *P. pingi*, F1 hybrids obtained from crossing female *Schizothorax wangchiachii* and male *P. pingi* exhibited significantly enhanced resistance, with hybrid fish being almost completely immune to infection [10]. These findings suggest that *P. pingi* possesses innate resistance to *I. multifiliis* and can serve as an excellent model for studying resistance mechanisms in fish. However, both crucian carp (*Carassius carassius*) [11] and yellow catfish (*Tachysurus fulvidraco*) [12] are highly susceptible to *I. multifiliis*, with yellow catfish being particularly vulnerable. The skin of yellow catfish is directly exposed, making it easier for the larvae (theronts) of the parasite to penetrate and infest [13]. Based on this, the present project selects *I. multifiliis*-susceptible scaled cyprinid fish (e.g., crucian carp) and scaleless siluriform fish (e.g., yellow catfish) for comparison with the naturally resistant *P. pingi*.

The skin and skin mucus are crucial materials for investigating the mechanisms of fish resistance against *I. multifiliis*. The skin acts as a vital mucosal surface and immune barrier in teleost fish, serving as the primary site for parasite invasion and establishment. Infection by *I. multifiliis* causes severe histopathological damage to the skin and mucosal tissues, which is a major contributor to host mortality [14]. Metabolomics and metagenomics are emerging tools for studying parasite-related diseases, holding great potential for drug and vaccine development [15]. Metabolomics and metagenomics can reveal the underlying mechanisms of host–parasite interactions by systematically analyzing changes in endogenous small molecule metabolites and microorganisms within an organism following parasitic infection. In Palaemon sinensis infected with *Tachaea chinensis*, the host enhances glycolysis and the tricarboxylic acid (TCA) cycle to release more energy in response to the infection pressure [16]. In a zebrafish model, the study found that salicylaldehyde, produced by the gut microbe Pelomonas, is a potent anti-parasitic agent capable of inhibiting egg hatching in *Pseudocapillaria tomentosa*, both in vitro and in vivo [17]. In grass carp infected with *Dactylogyrus lamellatus* in the gills, even though the infection site is localized to the gills, the composition of their gut microbiota undergoes significant alterations [18]. Acinetobacter was identified as the best biomarker for *Sparus aurata* parasitized by *Enteromyxum leei* [19]. Using untargeted metabolomics, we will investigate the metabolite composition and metabolic pathway characteristics in the skin tissue and mucus of *P. pingi*, aiming to reveal differential metabolites and pathways closely associated with its resistance. Through metagenomic sequencing, we will compare the composition and functional profiles of the skin (including mucus) microbiota of *P. pingi*, identifying key microbial communities and functions linked to its resistance against *I. multifiliis*. This study provides a foundation for breeding disease-resistant strains, offers significant theoretical and practical value for the prevention and control of ichthyophthiriasis, and promotes the development of new pharmaceuticals, vaccines, and diagnostic methods for combating this disease.

## 2. Materials and Methods

### 2.1. Sample Collection

*P. pingi* (average body weight 7.22 g) were collected from the fish propagation station of the Jinping-Guandi Hydropower Station on the Yalong River, while crucian carp (average body weight 7.32 g) and yellow catfish (average body weight 7.21 g) were obtained from a fry breeding base in Neijiang. All fish were no more than 4 months old, and no outbreak of *I. multifiliis* had occurred in the 4 months prior to sampling. Each fish was examined for ectoparasites to ensure they were healthy and free from parasitic infections. Gills, skin, eyes, and fins were inspected under a light microscope to confirm the absence of pathological lesions caused by pathogens. To minimize handling stress and injury, fish were acclimated for over 2 h and transferred using a submerged net method by trained personnel, with out-of-water time kept under 1 min, resulting in no observed physical damage throughout the study. Prior to the infection experiment, all tanks were thoroughly cleaned to eliminate potential pathogens and contaminants. The cleaning procedure involved scrubbing the tanks with a diluted bleach solution (1% sodium hypochlorite) followed by copious rinsing with tap water. To ensure the complete removal of any residual disinfectant that could harm the fish, the tanks were then treated with a sodium thiosulfate solution for neutralization and finally rinsed three times with dechlorinated water. To prevent cross-contamination, all tools used in the experimental procedures (including but not limited to nets, transfer buckets, etc.) were disinfected by immersion in a 50 mg/L sodium hypochlorite solution for 30 min before and after use. They were then thoroughly rinsed with sterile water and air-dried to ensure the absence of *I. multifiliis* or other potential contaminants. A total of 15 *P. pingi*, 15 crucian carp and 15 yellow catfish were reared separately in nine 300 L tanks (bottom area = 80 cm × 60 cm; height = 60 cm), with 5 fish per tank. This resulted in a stocking density of approximately 0.12~0.122 g/L and 10.4 fish per m^2^ of bottom area. All experimental fish were subjected to a 15-day quarantine and acclimation period in tanks before the start of the experiment [20]. During this time, their feeding, swimming, and body surface health status were observed and recorded daily to ensure the absence of any *I. multifiliis* infection or clinical signs of other diseases [21]. The fish were fed once daily (at 8:00) with commercial pellet feed at a rate of 2% of their body weight. All three fish species were fed the same commercial sinking pellet feed (Tongwei Co., Ltd., Chengdu, China) formulated to contain ≥32% crude protein, ≥5% crude fat, ≤8% crude fiber, ≤16% crude ash, ≤12% moisture, ≥1.0% total phosphorus, and ≥1.5% lysine on a dry matter basis, with 1.5 mm diameter pellets suitable for ingestion by the 7 g fish. The culture water was aerated tap water, maintained at a temperature of 22 ± 1.5 °C and dissolved oxygen ≥ 5.0 mg/L. One-third of the water was replaced daily (at 18:00) with fresh aerated tap water. During the rearing period, the light intensity and photoperiod were set at 1000 lx and 12L:12D, respectively. At the beginning of the comparative infection experiment, the water level in the tanks was reduced to one-third of the original volume, and 1.25 mL of *I. multifiliis* suspension (20,000 theronts/fish) was added to the tanks [9]. After 12 h of infection in darkness, the water level in the tanks was restored. On Day 5 post-infection, one fish was randomly selected from each tank and euthanized using 300 mg/L MS-222. The entire left-side skin was collected, rapidly frozen in liquid nitrogen, and sent to Novogene biotechnology company (Beijing, China) for testing.

### 2.2. Liquid Chromatography-Tandem Mass Spectrometry (LC-MS/MS) Analysis

Approximately 100 mg of skin sample, ground in liquid nitrogen, was placed in a microcentrifuge tube, and 500 μL of 80% aqueous methanol was added. The mixture was vortexed, allowed to stand in an ice bath for 5 min, and then centrifuged at 15,000× *g* and 4 °C for 20 min. A certain volume of the supernatant was diluted with mass spectrometry-grade water to achieve a methanol content of 53%. The mixture was centrifuged again at 15,000× *g* and 4 °C for 20 min, and the supernatant was collected for LC-MS/MS analysis [22]. The chromatographic conditions were as follows: column: Hypersil Gold column (C18); column temperature: 40 °C; flow rate: 0.2 mL/min; Positive ion mode (POS): mobile phase A: 0.1% formic acid, mobile phase B: methanol; Negative ion mode (NEG): mobile phase A: 5 mM ammonium acetate, pH 9.0, mobile phase B: methanol; scan range: *m*/*z* 100–1500. The ESI source settings were as follows: spray voltage: 3.5 kV; sheath gas flow rate: 35 psi; auxiliary gas flow rate: 10 L/min; ion transfer tube temperature: 320 °C; RF lens level: 60; auxiliary gas heater temperature: 350 °C; polarity; MS/MS secondary scanning was performed in data-dependent mode.

### 2.3. Data Preprocessing and Metabolite Identification

The raw data files were imported into CD 3.1 software for processing. Initial screening of each metabolite was performed based on parameters such as retention time and mass-to-charge ratio. Peak alignment across different samples was conducted with a retention time deviation of 0.2 min and a mass deviation of 5 ppm to enhance identification accuracy. Subsequently, peak extraction was performed with settings including a mass deviation of 5 ppm, signal intensity deviation of 30%, signal-to-noise ratio of 3, minimum signal intensity, and adduct ion information, while also quantifying peak areas. Target ions were integrated. Molecular formulas were predicted based on molecular ion peaks and fragment ions, followed by comparison against the mzCloud, mzVault, and Masslist databases. Background ions were removed using blank samples. The raw quantitative results were normalized using the formula: to obtain relative peak areas. Compounds with a coefficient of variation (CV) of relative peak area greater than 30% in Quality Control (QC) samples were excluded, resulting in the final identification and relative quantification of metabolites [23]. Data processing was performed on a Linux operating system (CentOS version 6.6) using R (version 4.0.3) and Python (version 3.7.4) software.

### 2.4. Statistical Analysis of Metabolomics Data

Identified metabolites were annotated using the KEGG, HMDB, and LIPIDMaps databases. For multivariate statistical analysis, the metabolomics data processing software metaX (version 1.4.16) [24] was used to transform the data, followed by Principal Component Analysis (PCA) and Partial Least Squares-Discriminant Analysis (PLS-DA) [25] to obtain the Variable Importance in Projection (VIP) value for each metabolite [26]. For univariate analysis, a t-test was used to calculate the statistical significance (*p*-value) of each metabolite between two groups [27], and the fold change (FC) was calculated. The default criteria for screening differential metabolites were VIP > 1, *p* < 0.05, and FC ≥ 1.5 or FC ≤ 0.667 [28,29,30]. Volcano plots were generated using the R package ggplot2 (version 3.3.5), integrating the three parameters VIP value, log2 (FoldChange), and −log10 (*p*-value) to screen metabolites. Cluster heatmaps were drawn using the R package Pheatmap (version 1.0.12), with metabolite data normalized using z-score. Violin plots between differential metabolites was performed using the vioplot() function in R (version 4.0.3). Venn diagrams was generated using the VennDiagram() function in R. Matchstick diagram and *p*-value bubble plots were generated using the R package ggplot2 (version 3.3.5). The KEGG database was used to study metabolite functions and metabolic pathways. A metabolic pathway was considered enriched when *p* < 0.01, and significantly enriched when *p* < 0.05.

### 2.5. DNA Extraction and PCR Amplification

Genomic DNA was extracted from samples using a DNA extraction kit (TIANGEN Biotech Co., Ltd., Beijing, China), with the primer amplification region targeting the 16S V3-V4 hypervariable region. All PCR mixtures contained 15 µL of Phusion High-Fidelity PCR Master Mix, 0.2 µM of each primer, and 10 ng of genomic DNA template. The PCR conditions were: initial denaturation at 98 °C for 1 min; 30 cycles of denaturation at 98 °C for 10 s, annealing at 50 °C for 30 s, and extension at 72 °C for 30 s; followed by a final extension at 72 °C for 5 min. PCR products were purified using magnetic beads. Based on the concentration of the PCR products, equal amounts were pooled, thoroughly mixed, and then detected to recover the target bands.

### 2.6. Library Construction, Sequencing, and Data Quality Control

The constructed libraries were quantified using Qubit (Thermo Fisher Scientific, Waltham, MA, USA) and qPCR (Bio-Rad Laboratories, Hercules, CA, USA). After confirming library quality, PE250 sequencing was performed on the NovaSeq600 platform (Illumina, Inc., San Diego, CA, USA). Based on barcode sequences and PCR amplification primer sequences, the sequencing data were demultiplexed into respective sample data. After removing barcodes and primer sequences, FLASH (Version 1.2.11) was used to assemble reads for each sample, resulting in Raw Tags [31]. Cutadapt (version 1.9.1) was then used to match the reverse primer sequence and trim the remaining sequence to prevent interference in subsequent analyses [32]. The assembled Raw Tags were subjected to strict filtering using fastp software (Version 0.23.1) to obtain high-quality Clean Tags [33]. The Tags obtained after the above processing required chimera removal. Tag sequences were compared against species annotation databases (Silva database for 16S/18S) to detect chimeric sequences, which were subsequently removed, yielding the final Effective Tags [34].

### 2.7. ASV Denoising and Taxonomic Annotation

For the obtained Effective Tags, denoising was performed using the DADA2 module [35] in QIIME2 (Version 2022.02) to obtain the final Amplicon Sequence Variants (ASVs) and feature table [36]. Species annotation was performed using QIIME2 software with the Silva 138.1 database [37]. Rapid multiple sequence alignment was performed using QIIME2 software to obtain the phylogenetic relationships of all ASV sequences. Finally, the data for each sample were normalized based on the sample with the smallest data volume. Subsequent alpha diversity and beta diversity analyses were based on the normalized data. Based on the top 10 abundant species at each taxonomic level (phylum, class, order, family, genus, species) in each sample, distribution histograms of relative abundance were plotted using the SVG function in Perl. Heatmaps were generated using the abundance information of the top 35 abundant species at each taxonomic level to visually display differences in abundance and taxon clustering, implemented using the pheatmap() function in R. Ternary plots for the top 10 taxa at each taxonomic level can be used to display abundance differences among three samples, calculated using the vcd() function in R.

### 2.8. Sample Complexity Analysis and Functional Prediction

The Chao1 was calculated using QIIME2 software. To assess the complexity of community composition and compare differences between groups, beta diversity analysis based on weighted and unweighted distances was performed in QIIME2. Weighted and unweighted Unifrac beta diversity distances were calculated using QIIME2 software. A clustering tree was then drawn to display the Unifrac distances between samples, implemented using Perl. Based on the weighted Unifrac distance matrix, a clustering tree was constructed using UPGMA. The UPGMA tree was plotted using the UPGMA.tre function in QIIME2. Principal Coordinates Analysis (PCoA) was used to obtain principal coordinates from complex, multidimensional data and visualize them. The distance matrix of weighted or unweighted Unifrac distances between samples was obtained before transformation to a new set of orthogonal axes. PCoA analysis was calculated and plotted using the ade4 (version 1.7-16) and ggplot2 packages (version 3.3.5) in R software (version 4.0.3). Functional prediction analysis was performed using Tax4Fun (V0.3.1).

## 3. Results

### 3.1. Metabolite Classification and Grouping Status

**Figure 1:**
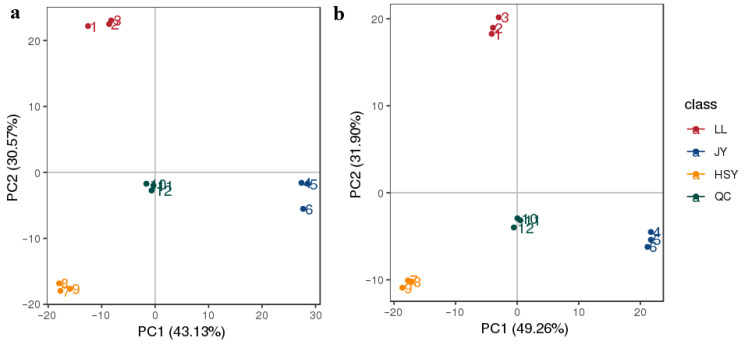
PCA of the total sample. (**a**) Positive ion mode; (**b**) Negative ion mode; LL: *P. pingi*; JY: Crucian carp; HSY: Yellow catfish; QC: Quality Control; The same below.

A total of 742 metabolites in POS and 441 metabolites in NEG were identified from the nine samples in this study. Statistics on the chemical classification of the identified metabolites revealed that the top five categories in POS were: Lipids and lipid-like molecules (40.94%), Organic acids and derivatives (20.60%), Organoheterocyclic compounds (14.64%), Benzenoids (7.44%), and Organic nitrogen compounds (3.97%) (Figure S1). In NEG, the top five categories were: Lipids and lipid-like molecules (50.16%), Organic acids and derivatives (20.50%), Organoheterocyclic compounds (7.89%), Nucleosides, nucleotides, and analogs (7.26%), and Organic oxygen compounds (5.68%) (Figure S2). PCA was performed on the peaks extracted from all experimental samples and QC samples. Smaller differences between QC samples indicate better method stability and higher data quality, which is visually represented on the PCA score plot by the clustering of QC samples. The samples from *P. pingi*, crucian carp, yellow catfish, and the QC samples each formed distinct clusters (Figure 1), demonstrating the high quality of the data used in this study and supporting the accuracy of subsequent analyses.

### 3.2. Screening of Differential Metabolites

**Figure 2:**
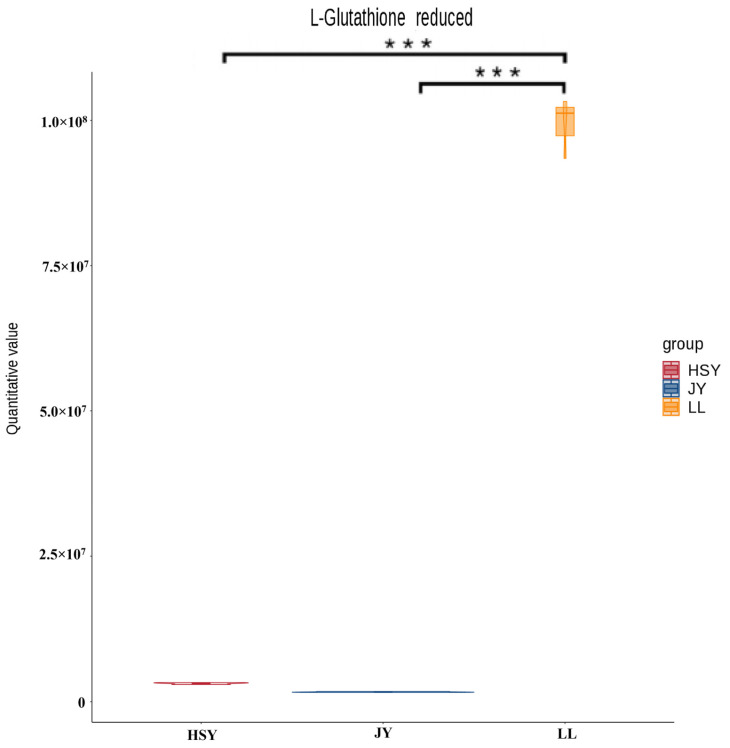
The expression levels of L-Glutathione reduced compounds in HSY, JY and LL groups under positive ion mode. Violin plots with *p*-values of L-Glutathione reduced. “***” indicates a significant difference between groups (*p* < 0.0005).

In positive ion mode, 306 significantly differential metabolites were identified in *P. pingi* compared to crucian carp, with 119 upregulated and 187 downregulated (Figure S3); 238 significantly differential metabolites were identified in *P. pingi* compared to yellow catfish, with 93 upregulated and 145 downregulated (Figure S4). In negative ion mode, 196 significantly differential metabolites were identified in *P. pingi* compared to crucian carp, with 77 upregulated and 119 downregulated (Figure S5); 171 significantly differential metabolites were identified in *P. pingi* compared to yellow catfish, with 71 upregulated and 100 downregulated (Figure S6). Many of the metabolites upregulated in *P. pingi* are related to antioxidant processes. For example, L-Glutathione reduced is one of the most important antioxidants in cells, scavenging free radicals and protecting cells from oxidative damage [38]. In positive ion mode, the content of L-Glutathione reduced in the skin tissue of *P. pingi* was 31 times that in yellow catfish and 59 times that in crucian carp (Figure 2).

**Figure 3:**
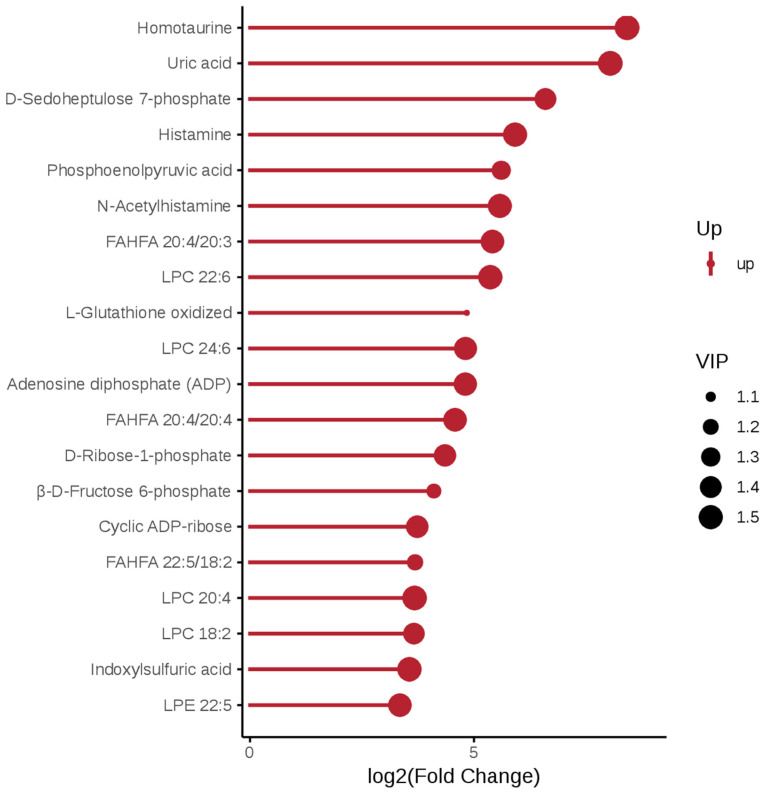
Matchstick diagram of differential metabolites between the LL and JJ groups under negative ion mode. Take the Fold Change value of the differential metabolites as the base 2 for logarithmic transformation, and sort them in this order. Select the top 20 metabolites with upregulation for each to display the matchstick chart, with red representing upregulation. The length of the rod represents the size of log2(Fold Change); The size of the dot represents the size of the VIP value, the same below.

**Figure 4:**
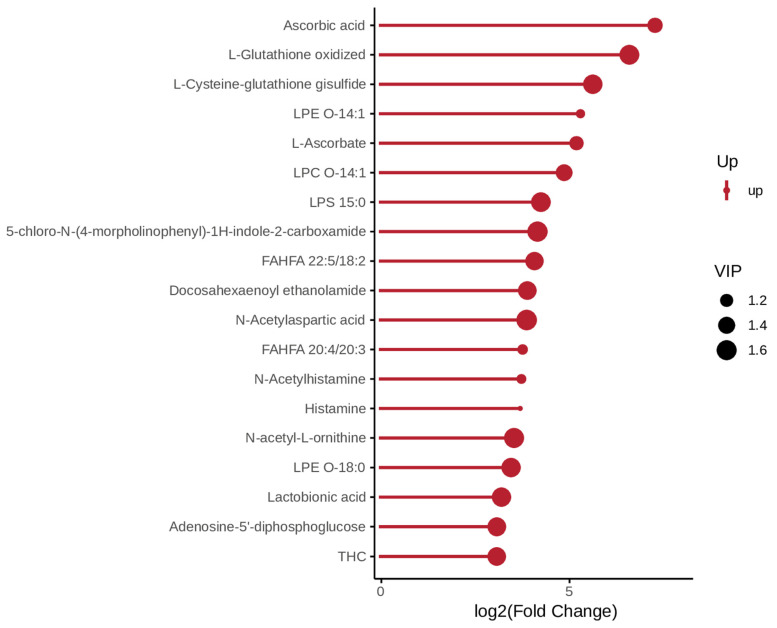
Matchstick diagram of differential metabolites between the LL and HSY groups under negative ion mode.

In negative ion mode, the metabolites upregulated in the *P. pingi* and crucian carp comparison were associated with oxidative stress level (L-Glutathione oxidized and Uric acid) and immune/allergic response (Histamine and N-Acetylhistamine) (Figure 3). In the comparison group of *P. pingi* and yellow catfish, the top three metabolites that were upregulated were Ascorbic acid, L-Glutathione oxidized and L-Cysteine-glutathione gisulfide, which mainly involved antioxidants (Figure 4).

### 3.3. Functional Enrichment Analysis of Differential Metabolites

**Figure 5:**
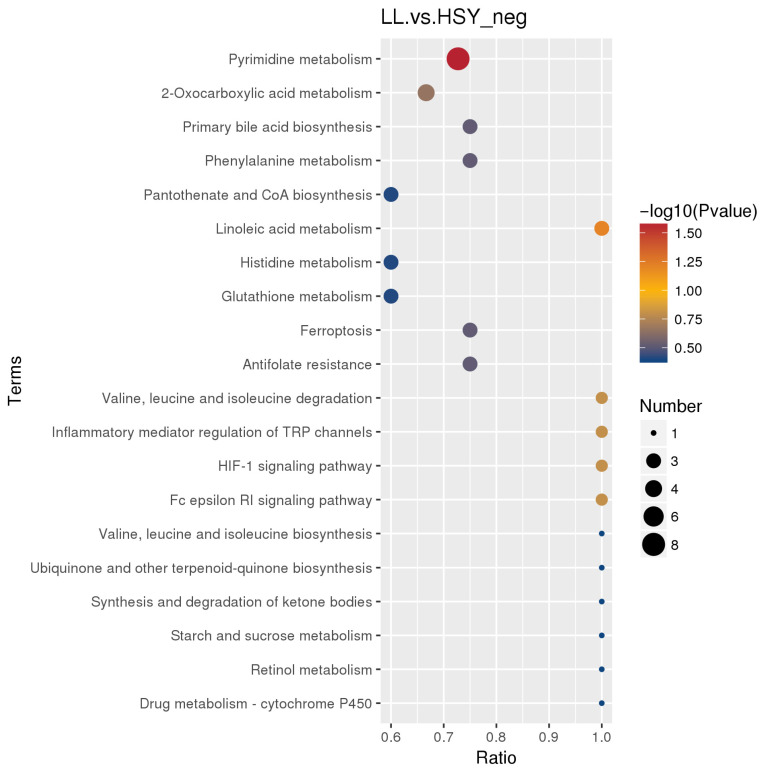
KEGG rich distribution point plots of the differential metabolites in LL and HSY groups in negative ion mode. The horizontal axis represents the ratio of the number of differentially expressed genes annotated onto the KEGG pathway to the total number of differentially expressed genes, and the vertical axis represents the KEGG pathway.

Functional enrichment analysis was performed on the differential metabolites. In negative ion mode, the pathways involving differential metabolites between *P. pingi* and yellow catfish encompassed several important biological processes potentially associated with the resistance of *P. pingi* to *I. multifiliis* (Figure 5). The first major category consisted of pathways related to oxidative stress and antioxidant defense, centered on Glutathione metabolism (map00480) and also including Drug metabolism-cytochrome P450 (map00982) and Retinol metabolism (map00830). The second major category comprised pathways associated with immune and inflammatory responses, such as the Fc epsilon RI signaling pathway (map04664), Inflammatory mediator regulation of TRP channels (map04750), and Histidine metabolism (map00340).

### 3.4. Changes in the Composition of the Skin Microbiota

**Figure 6:**
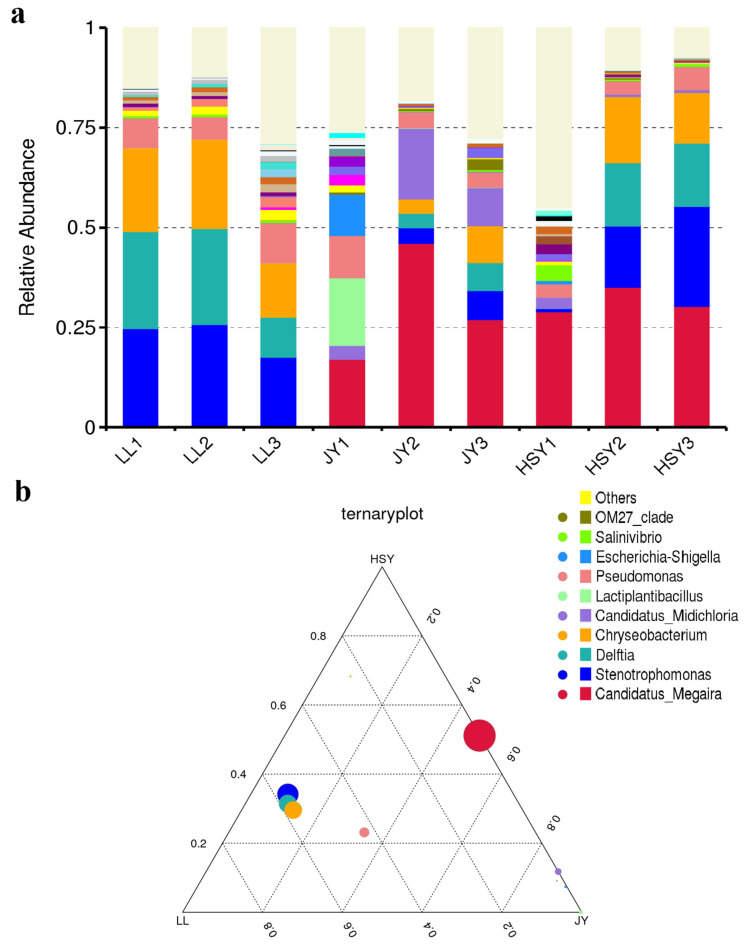
Top10 Bar chart (**a**) and ternaryplot (**b**) of relative abundance of species (Genus). In the ternaryplot, the three vertices represent three groups, and the circles represent species. The size of the circles is directly proportional to their relative abundance. The closer the circle is to a certain vertex, the higher the content of this species in that group.

After filtering Raw Tags for low quality, short length, and chimeras, the final Tag sequences used for subsequent analysis were 93,861 (LL1), 91,925 (LL2), 79,130 (LL3), 100,163 (JY1), 85,557 (JY2), 91,194 (JY3), 95,853 (HSY1), 90,573 (HSY2), and 99,046 (HSY3), respectively (Table S1). The Q20 of the data exceeded 98%, and the Q30 exceeded 94%, indicating good data quality suitable for subsequent analysis. Based on the species annotation results at different taxonomic levels, the top 10 species with the highest relative abundance in each sample or group at each taxonomic level (Phylum, Class, Order, Family, Genus, Species) were selected. The remaining species were categorized as “Others” to generate relative abundance bar plots illustrating the species annotation results for each sample across different taxonomic levels. At the genus level, the results showed that, compared to crucian carp and yellow catfish, *P. pingi* lacked species belonging to the genus *Candidatus_Megaira* (Figure 6a). *Candidatus_Megaira* is a group of obligate intracellular parasitic bacteria, commonly known as symbionts or parasites of freshwater algae and protists [39]. *C. Megaira* is widely present in *I. multifiliis. I. multifiliis* may be dependent on this endosymbiotic relationship, and the association between *C. Megaira* and *I. multifiliis* is more diverse than previously thought [40]. Ternaryplot shows that the *C. Megaira* genus was present in high concentrations in the crucian carp and yellow catfish groups, while the *Stenotrophomonas*, *Delftia* and *Chryseobacterium* genera were present in high concentrations in *P. pingi* group (Figure 6b). The Venn diagram shows that there were 102 common characteristic sequences among *P. pingi*, crucian carp and yellow catfish. *P. pingi* has the most unique characteristic sequences, with 1653 (Figure S7).

### 3.5. Changes in α- and β-Diversity of Skin Microbiota

**Figure 7:**
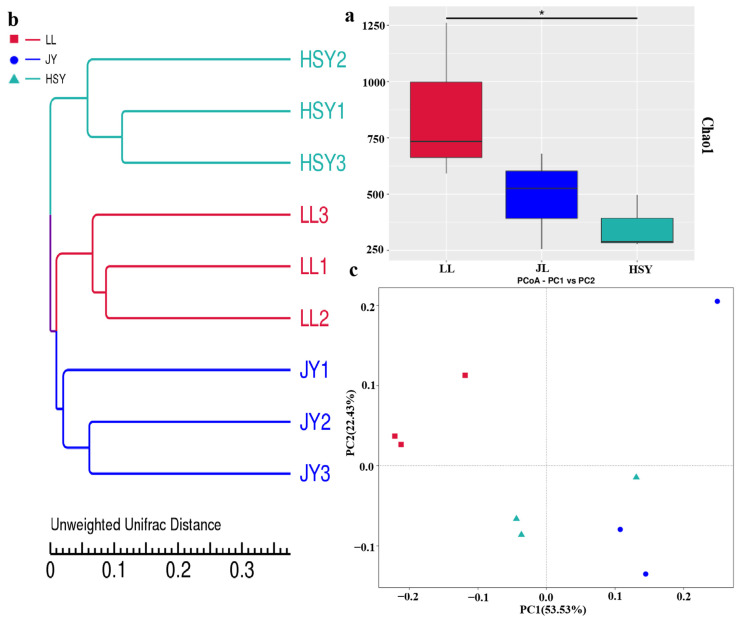
Alpha diversity and beta diversity of differences groups. Alpha-diversity included chao1 (**a**). “*” indicates a significant difference between groups (*p* < 0.05). Beta diversity included PCoA UPGMA clustering tree (**b**) and diagram (**c**). The abscissa represents one principal component, the ordinate represents the other principal component, and the percentage represents the value of the principal component’s contribution to the sample difference.

The Chao1 index directly estimates the number of species detected. The index was higher for *P. pingi* than for crucian carp and significantly higher than for yellow catfish, indicating a greater number of microbial species on the skin of *P. pingi* compared to both crucian carp and yellow catfish (Figure 7a). UPGMA cluster analysis based on the Unweighted Unifrac distance matrix showed that the crucian carp and yellow catfish groups clustered together on one branch (Figure 7b). PCoA analysis also revealed that the crucian carp and yellow catfish groups were closer in distance, while the *P. pingi* group formed a distinct cluster located further away (Figure 7c). This indicates that the microbial community composition on the skin of crucian carp and yellow catfish is more similar to each other and distinct from that of *P. pingi*.

### 3.6. Genus with Significant Differences Between Groups

**Figure 8:**
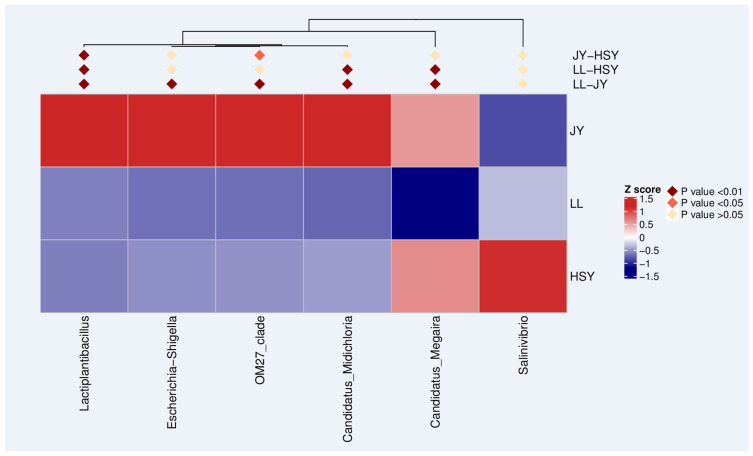
Clustering heat map of differential genus. Z-score = (Abundance of the species in a sample—average abundance of the species in all samples)/standard deviation of the abundance of the species in all samples. Red: indicates that the species is significantly enriched in the corresponding sample (Z-score > 0); blue: indicates that the species is significantly absent in the corresponding sample (Z-score < 0); white: indicates that the species is at the average level in the corresponding sample (Z-score = 0), the same below.

The *p*-values were obtained by conducting hypothesis testing on inter-group species abundance data using the MetagenomeSeq method. Species with statistically significant differences between groups were screened based on the *p*-values, and a heatmap was plotted to show the abundance distribution of these differential species across groups. Compared to crucian carp and yellow catfish, the genera such as *Lactiplantibacillus*, *Escherichia-Shigella*, *OM27_clade*, *C. Megaira*, *C. Midichloria*, and *Salinivibrio* were lower in *P. pingi*. The pathogenic bacterium *Escherichia-Shigella* was significantly more abundant in the skin of crucian carp than in bass (*p* < 0.01), while the parasitic bacteria *C. Megaira* and *C. Midichloria* were highly significantly more abundant in the skin of yellow catfish and crucian carp than in bass (*p* < 0.01) (Figure 8).

### 3.7. Functional Enrichment Analysis

**Figure 9:**
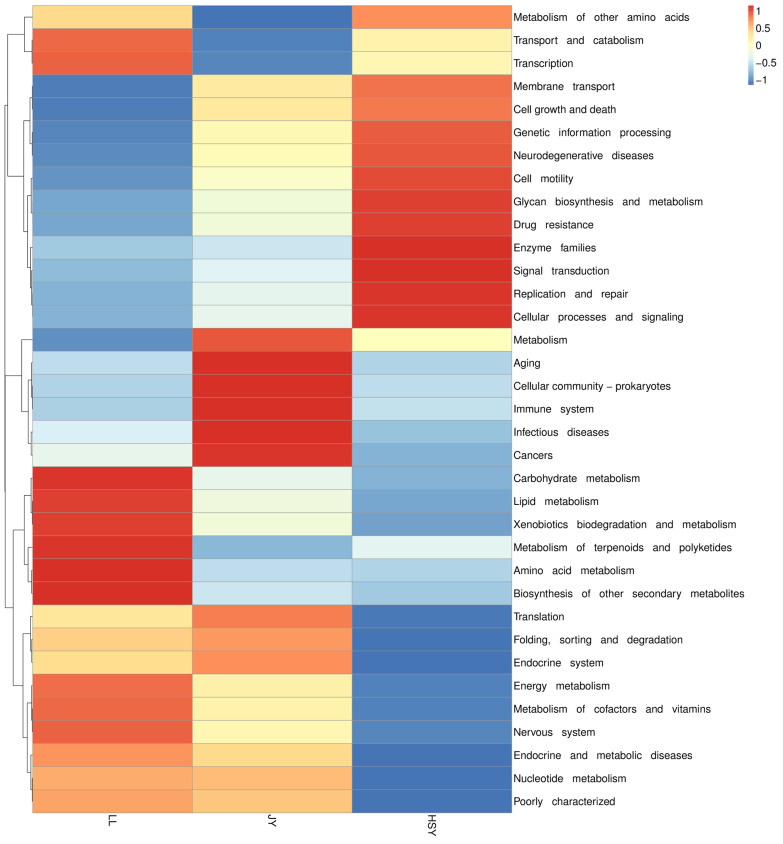
Tax4Fun functional annotation clustering heat map of the skin microbiota in LL-JY-HSY groups. The vertical axis represents sample information, while the horizontal axis represents functional annotation information. The clustering tree on the left side of the figure is a functional clustering tree. The values in the heatmap correspond to the Z-scores obtained after normalizing the relative abundance of each functional category per row.

Tax4Fun functional annotation and relative abundance clustering analysis revealed that the pathways enriched in *P. pingi* were primarily associated with the metabolism of various complex compounds. These included carbohydrate metabolism, lipid metabolism, xenobiotics biodegradation and metabolism, metabolism of terpenoids and polyketides, amino acid metabolism, biosynthesis of other secondary metabolites, energy metabolism, metabolism of cofactors and vitamins, and nucleotide metabolism. In contrast, crucian carp and yellow catfish showed significant enrichment in pathways related to various diseases and immune functions, such as neurodegenerative diseases, drug resistance, aging, immune system, infectious diseases, and cancers (Figure 9).

## 4. Discussion

### 4.1. Integrated Antioxidant Defense as a Hallmark of Disease Resistance

When facing parasitic invasion, the antioxidant system in fish collaborates closely with the immune system to form a crucial defensive barrier [41]. Two extracellular protozoa primarily induce ichthyophthiriasis in fish: the freshwater-dwelling *I. multifiliis* and its marine counterpart, *Cryptocaryon irritans* [42]. Research on resistant species like the golden rabbitfish (*Siganus oramin*) has revealed that an efficient antioxidant response is a key component of their defense strategy. These fish not only regulate and clear excess reactive oxygen species (ROS) effectively to mitigate tissue damage but also employ specific anti-parasitic proteins, such as L-amino acid oxidase (LAAO), for direct action against *C. irritans* [43,44]. Conversely, in susceptible species like the large yellow croaker (*Larimichthys crocea*), infection by *C. irritans* induces systemic oxidative stress, characterized by a significant decrease in hepatic glutathione peroxidase (GSH-Px) activity, a sharp increase in superoxide dismutase (SOD), and a peak in the oxidative damage marker malondialdehyde (MDA) within hours [45]. Similarly, infection of *Coris julis* by *Scaphanocephalus* sp. triggers the activation of antioxidant enzymes (SOD, CAT, glutathione S-transferase) in mucosal tissues, underscoring their role as a first line of defense [46]. Taken together, these comparative studies from the literature consistently highlight that the capacity to mount a robust, pre-emptive antioxidant response, rather than just a reactive one, may be a key factor differentiating resistant from susceptible species [42,43,44,45,46]. This well-established paradigm supports a plausible hypothesis for our own results: the differential susceptibility we observed among *P. pingi*, crucian carp, and yellow catfish could similarly be underpinned by variations in their capacity to manage oxidative stress during infection. Future studies directly measuring antioxidant enzyme activity would be valuable to confirm this mechanism in our model system.

*I. multifiliis* invasion induces intense oxidative stress in the host, and such infection causes tissue damage via lipid peroxidation in susceptible species like silver catfish [47]. The constitutive enrichment of reduced glutathione in *P. pingi* would enable the rapid neutralization of parasite-induced ROS bursts, protecting skin cells from oxidative damage and potentially directly interfering with parasite establishment. In contrast to *P. pingi*, the susceptible species (crucian carp and yellow catfish) exhibited drastically lower levels of reduced glutathione that was tens of times less abundant. This deficiency suggests a severely limited capacity to mount an immediate, pre-emptive defense against the parasite-induced oxidative burst, leaving skin tissues vulnerable to damage. Instead, the upregulation of metabolites related to immune and inflammatory responses, such as histamine, in these susceptible species indicates a shift towards a costly and potentially detrimental reactive state only after infection is established. The maintenance of a high antioxidant “baseline” may represent a proactive energy investment that pre-empts the high cost of an inflammatory immune response, which is characteristic of the susceptible species in our study. This aligns with the concept of “disease tolerance”—a defense strategy that minimizes fitness costs by maintaining host fitness during infection, as opposed to merely resisting pathogen burden. From a translational perspective, our findings move beyond merely identifying glutathione as a biomarker. They illuminate a fundamental physiological principle for breeding strategies: selecting for individuals with a constitutively robust antioxidant system may be more effective than selecting based on response to infection. This proactive defense mechanism, once entrenched in a breeding population, could provide broad-spectrum resilience against various stressors, not limited to *I. multifiliis* but potentially other diseases involving oxidative stress. Finally, while our data strongly point to the critical role of reduced glutathione, the integrated nature of the metabolic network we uncovered suggests that its efficacy is amplified by complementary pathways. The co-enrichment of cytochrome P450 and retinol metabolism indicates a sophisticated system for detoxifying lipid peroxidation products and repairing epithelial integrity. Therefore, the resilience of *P. pingi* is likely not attributable to a single metabolite but to the synergistic effect of a pre-configured, multi-layered chemical defense regime.

### 4.2. The Protective Skin Microbiome as an Ecological Barrier

The microbiota inhabiting the skin, gills, and gut of fish forms a complex ecosystem that serves as a critical first line of defense against parasitic invasions [48]. A common consequence of parasitic infection is the disruption of this microbial equilibrium. For instance, in yellowtail kingfish (*Seriola lalandi*) infected with the intestinal parasite *Enteromyxum leei*, gut microbiota structure is significantly altered, with a marked increase in *Proteobacteria* and a decrease in *Firmicutes* and *Actinobacteria*, a shift correlated with disease severity [49]. Similarly, gill infection in grass carp leads to reduced gut microbial diversity and specific taxonomic changes [50], illustrating a trans-organ microbial dysbiosis in response to localized parasitic challenge. Our microbiome analysis adds a critical dimension to this understanding, revealing that *P. pingi* possesses a skin microbial community fundamentally distinct from that of susceptible species. The higher α-diversity (Chao1 index) and unique β-diversity indicate a richer, more stable, and potentially more resilient microbial ecosystem, which is often associated with host health. The most indicative finding was the significant depletion of the parasitic bacteria *C. Megaira* and *C. Midichloria* on *P. pingi* skin. In contrast, the skin microbiome of crucian carp and yellow catfish exhibited a convergent and potentially disadvantageous structure. Their lower microbial diversity and closer clustering in beta-diversity analyses point to a less resilient microbial community. Crucially, both species showed significantly higher abundances of *C. Megaira* and *C. Midichloria*, which are associated with *I. multifiliis* itself. The presence of these symbionts on susceptible hosts may facilitate the establishment and survival of the parasite. Given that *C. Megaira* is an obligate intracellular parasite of freshwater ciliates [39,51], its high abundance on susceptible hosts may indicate a permissive microenvironment for ciliate-related parasites or may even involve complex tripartite interactions. The widespread presence of *C. Megaira* in *I. multifiliis* suggests that the two may have established a dependent relationship [40]. *C. Megaira* likely utilizes glycogen/glucose derived from its host [52], and *I. multifiliis* may have undergone further genome reduction as a result of forming a mutualistic symbiotic relationship with *C. Megaira* [16]. Prokaryotic endosymbionts have been identified in more than 200 species of ciliates [17], and these symbionts can provide hosts with nutritional support [53], defense mechanisms [54], or access to more favorable environments [55]. We hypothesize that the skin of susceptible fish, by hosting these parasitic bacteria, might be “primed” or ecologically modified in a way that facilitates the recognition, attachment, or development of *I. multifiliis*, which is itself a ciliate. Conversely, the absence of these specific parasitic bacteria on *P. pingi* skin likely renders it an “unfriendly” or unsuitable habitat, effectively eliminating this potential initial risk factor. This notion of a protective microbiome is further supported by functional predictions: the enrichment of fundamental metabolic pathways in *P. pingi*’s microbiota suggests a community in a stable, homeostatic state, whereas the skew towards disease and immune pathways in susceptible species implies a community under constant stress or in a dysbiotic, “immune-ready” state, which may be less effective at providing colonization resistance. In conclusion, the susceptibility of crucian carp and yellow catfish appears to be a consequence of a “double jeopardy”: an inherent weakness in the frontline antioxidant defense system coupled with a skin microbiome that is less diverse and enriched with pro-parasitic entities. Therefore, modulating the fish microbial environment through probiotics, prebiotics, or functional feeds represents a highly promising ecological approach for parasitic disease prevention, moving beyond purely chemical or immunological interventions [56].

### 4.3. Future Perspectives

However, a primary limitation of this study is its correlative nature. While we propose robust mechanistic hypotheses, they require direct experimental validation. Future work should include in vitro assays to verify the direct parasiticidal effect of GSH on *I. multifiliis* theronts and microbial transplantation experiments (e.g., co-housing or fecal microbiota transplant) to conclusively demonstrate the protective function of the *P. pingi*-associated microbial community. Furthermore, the roles of the host genetic background and specific immune factors (e.g., mucosal immunoglobulin and T-cell responses) in shaping and interacting with these observed metabolic and microbial phenotypes warrant deeper investigation using transcriptomic and immunological approaches. Understanding these complex interactions will be pivotal for developing comprehensive strategies to enhance disease resistance in aquaculture.

## 5. Conclusions

In conclusion, *P. pingi* resists *I. multifiliis* through the combined action of its skin’s antioxidant system and microbiome. High levels of antioxidants like glutathione provide a molecular defense, while a unique and diverse skin microbiome, lacking specific parasite-favoring bacteria, creates an unfavorable environment for the parasite. These insights offer a new foundation for developing disease-resistant fish breeds and eco-friendly control strategies, such as probiotic feeds, in aquaculture.

## Data Availability

All data generated and analyzed during this study are included in the published article.

## Supplementary Materials

The following supporting information can be downloaded at: https://www.mdpi.com/article/10.3390/biology14111546/s1, Figure S1: Metabolite Classification Pie Chart (positive ion mode); Figure S2: Metabolite Classification Pie Chart (negative mode); Figure S3: Volcanic Map (positive ion mode) of Differential Metabolites between *P. pingi* and crucian carp; Figure S4: Volcanic Map (negative mode) of Differential Metabolites between *P. pingi* and crucian carp; Figure S5: Volcanic Map (positive ion mode) of Differential Metabolites between *P. pingi* and yellow catfish; Figure S6: Volcanic Map (negative mode) of Differential Metabolites between *P. pingi* and yellow catfish; Figure S7: Comparison of three groups of feature sequences; Table S1. Statistical results of metagenomic sequencing data.
