# Supplementary material for: Metabolome and Metagenome Signatures Underlying the Differential Resistance of Percocypris pingi, Crucian Carp, and Yellow Catfish to Ichthyophthirius multifiliis Infection
Source: Biology (Basel). 2025 Nov 4;14(11):1546. doi: 10.3390/biology14111546 (PMC12650438; doi:10.3390/biology14111546)
Supplement: Supplementary file 1 [file biology-14-01546-s001.zip › biology-3946919-supplementary.pdf]

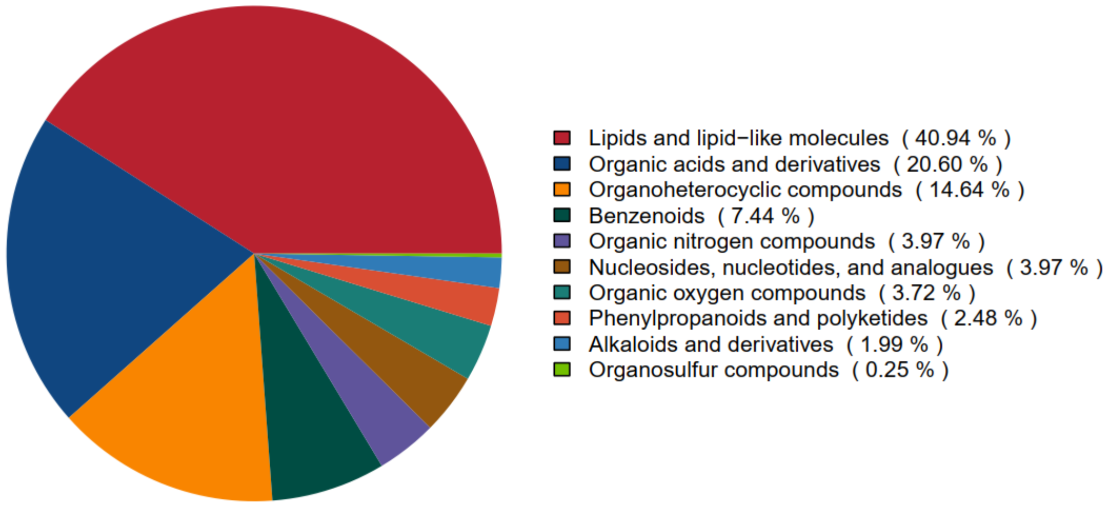

Figure S1. Metabolite Classification Pie Chart (positive ion mode)

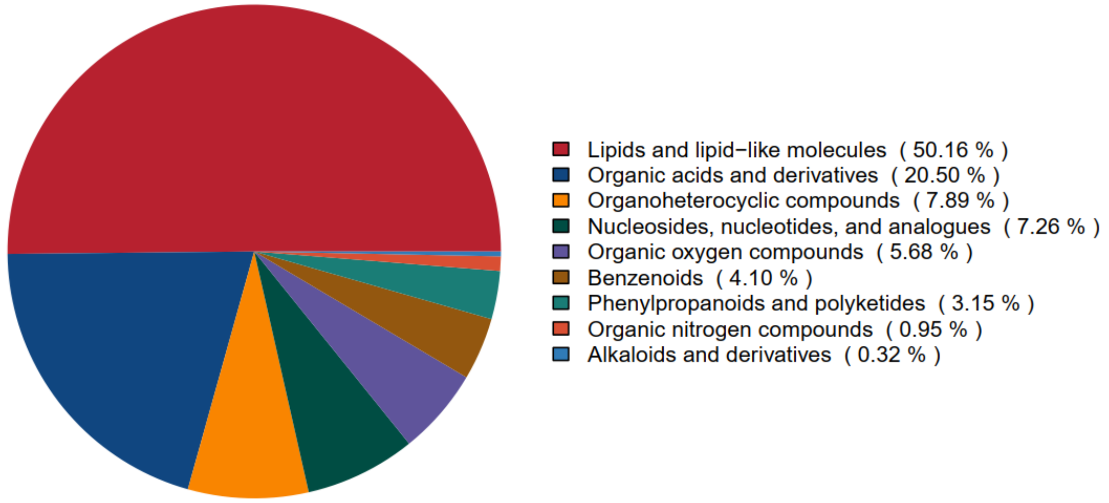

Figure S2. Metabolite Classification Pie Chart (negative mode)

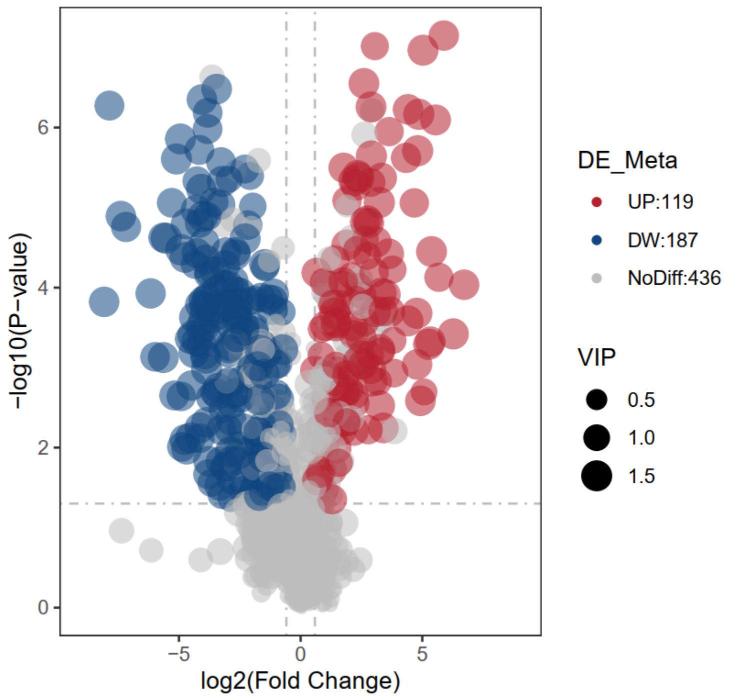

Figure S3. Volcanic Map (positive ion mode) of Differential Metabolites between *P. pingi* and crucian carp. The horizontal coordinate represents the log2FoldChange value, the vertical coordinate is -log10padj, and the dotted line indicates the threshold line of the differential gene screening criteria.

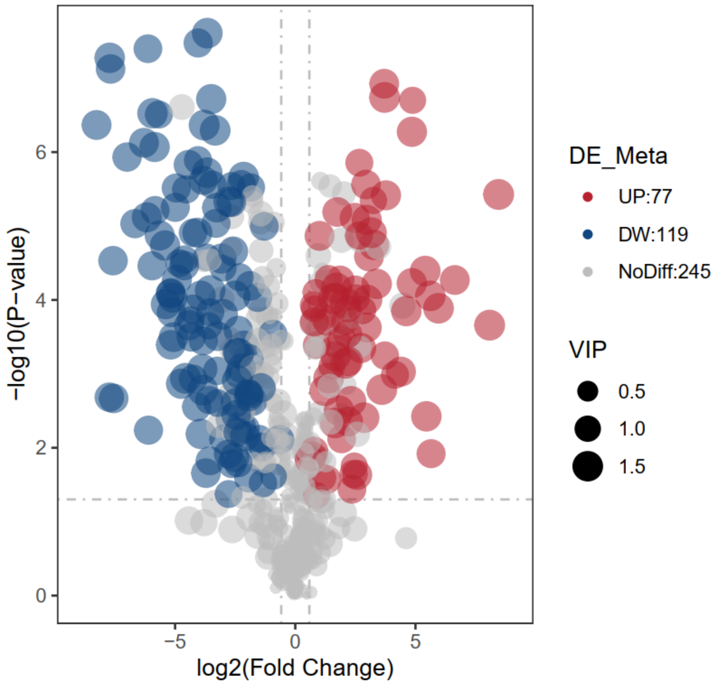

Figure S4. Volcanic Map (negative mode) of Differential Metabolites between *P. pingi* and crucian carp

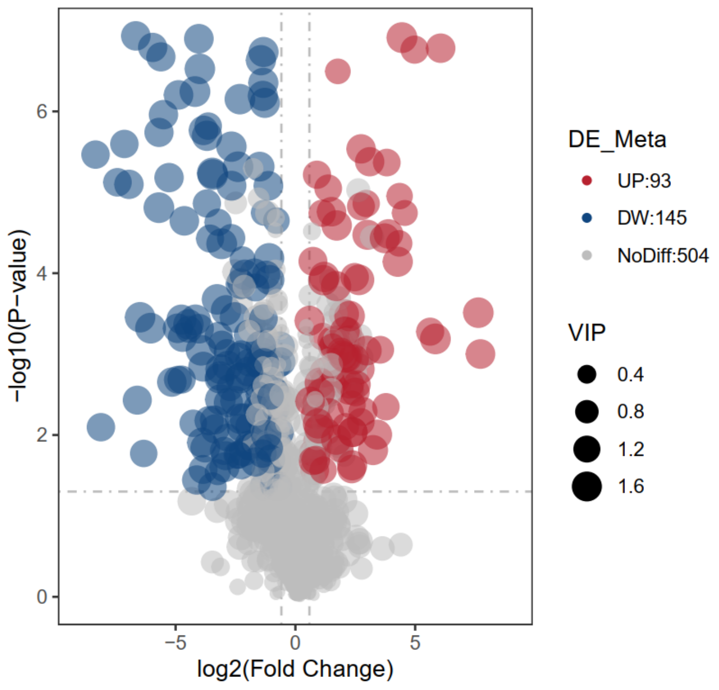

Figure S5. Volcanic Map (positive ion mode) of Differential Metabolites between *P. pingi* and yellow catfish

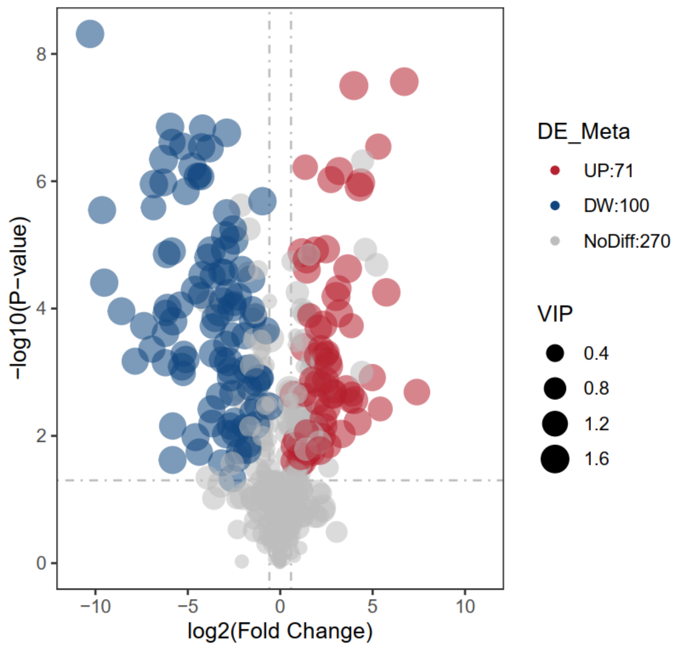

Figure S6. Volcanic Map (negative mode) of Differential Metabolites between *P. pingi* and yellow catfish

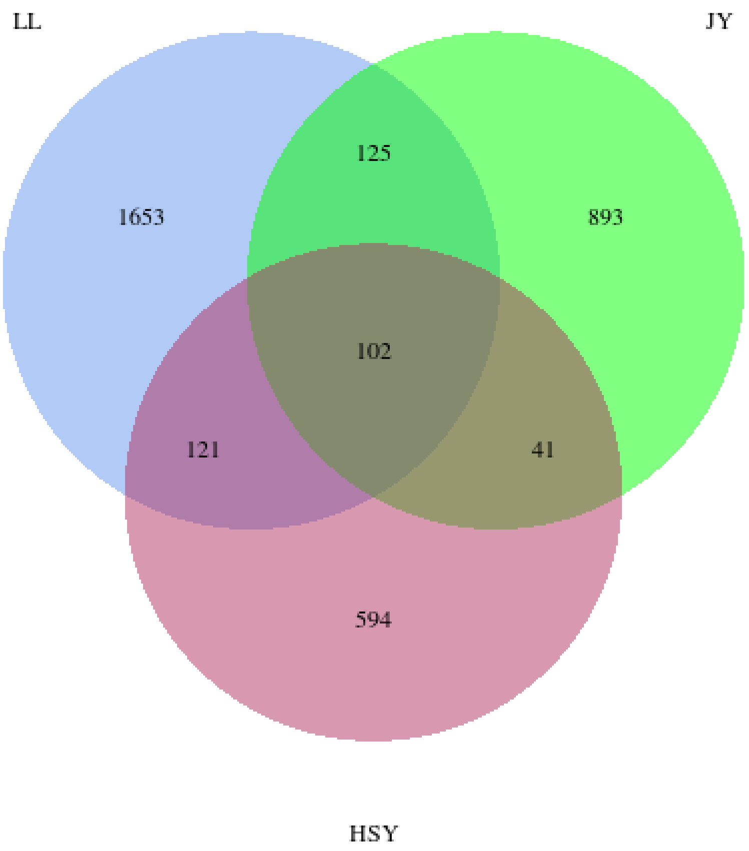

Figure S7. Comparison of three groups of feature sequences

Table S1. Statistical results of metagenomic sequencing data

Sample	RawPE[1]	Nochime[2]	Base(nt)[3]	Avglen(nt)[4]	GC[5]	Q20[6]	Q30
LL1	104076	93861	39944323	425.57	52.42%	98.64%	95.14%
LL2	104889	91925	39137003	425.75	52.34%	98.73%	95.43%
LL3	103888	79130	33480354	423.11	52.92%	98.69%	95.42%
JY1	104558	100163	42050609	419.82	52.21%	98.48%	94.82%
JY2	103969	85557	35153355	410.88	52.03%	98.70%	95.23%
JY3	103194	91194	37896091	415.55	52.21%	98.81%	95.54%

HSY1	106542	95853	39762019	414.82	52.43%	98.71%	95.39%
HSY2	104497	90573	37833067	417.71	51.92%	98.58%	94.99%
HSY3	111315	99046	41585394	419.86	52.00%	98.72%	95.37%

[1] rawPE represents the original PE reads taken off the machine; [2] Nochime is the tag sequence that is ultimately used for subsequent analysis after filtering chimeras, that is, Effective Tags; [3] Base is the number of bases in the final Effective Tags; [4] AvgLen is the average length of Effective Tags; [5] GC(%) represents the content of GC bases in Effective Tags; [6] Q20 and Q30 represent the percentages of bases with mass values greater than 20 (sequencing error rate less than 1%) and 30 (sequencing error rate less than 0.1%) in Effective Tags.
